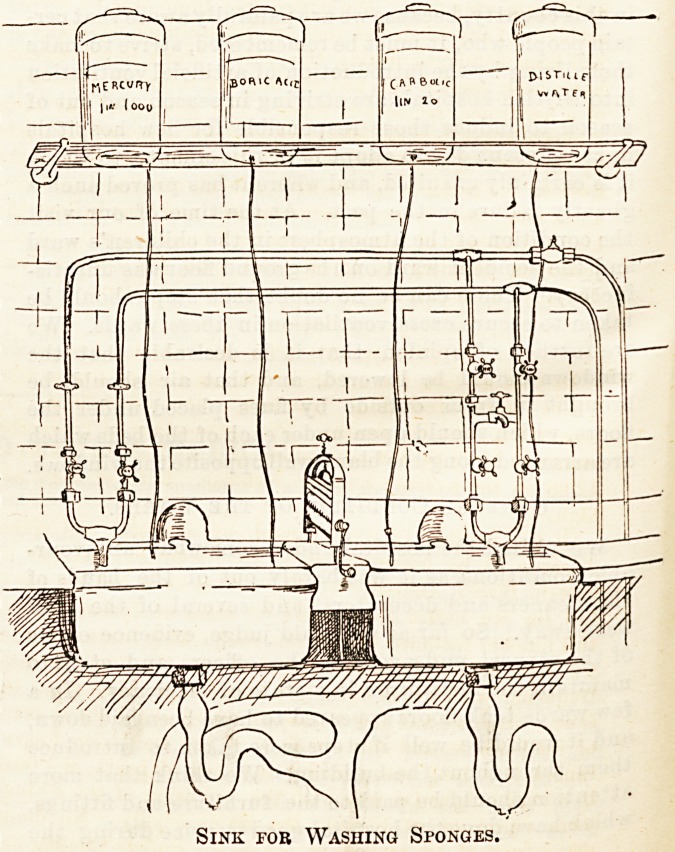# New Operating Theatre, the Middlesex Hospital

**Published:** 1895-08-31

**Authors:** 


### PRACTICAL DEPARTMENTS.

### NEW OPERATING THEATRE, THE MIDDLESEX HOSPITAL (*continued*).

The various fittings in the new theatre are the most modern and best of their kind, and no pains have been spared in arranging them. The shelves and tables for instruments, dressings, &c., are everywhere of glass, the former made to slide easily for cleaning purposes. They have been supplied by Messrs. Maw, Son, and Thompson. The dressers' stands are of both square and curved shapes, mounted on rubber castors. The Dent and Helyer's sinks are constructed on a novel principle. Hot and cold water is supplied through one tap, which is worked by the foot from beneath by pressure, as shown in the accompanying sketch. The supply is thus easily regulated, and all touching of taps by the hand is obviated. The sponge-washing and wringing arrangement is particularly excellent, and of this also we give a drawing, by Mr. Melhardo's permission. The sponges are washed in the left hand sink, passed through the wringer fixed between the two and rinsed again in the further basin. There are separate supplies of hot and cold water for other purposes.

Over this sink are large glass jars containing mercury, boric acid, carbolic, &c., each with its tubing.

The electric lighting has been carried out under the direction of the resident electrician, to whose invention many clever contrivances are directly due. The large centre light is a triumph of moveability, swinging right across the theatre by means of a pulley arrangement, which makes it possible for this powerful lamp to be placed literally in any position most convenient to the operating surgeon. It pulls down to the ground or can be raised above the head, and tilted and turned as convenience directs. A similar lamp is also fixed in the smaller operating-room, and there is in addition a most efficient supply of other lights at convenient angles. Any failure of light, though this is of the rarest occurrence anywhere in the Middlesex Hospital, so we are told, is guarded against by it being arranged on three circuits. The

Dent and Helyer's Sink.

anæsthetising room as well as all the other rooms are amply lighted.

The flooring throughout is of terazzo, quite the best material for the purpose, and the wood work of the student gallery, &c., is of teak. It would be difficult to find any room

Sink for Washing Sponges.

for criticism in a work so well planned and arranged as is this latest addition to the Middlesex Hospital. It may well claim to be a "model" theatre, and certainly stands superior to similar departments in any other London hospital. Indeed, in the matter of keeping up to date in all essential appliances, the Middlesex Hospital generally sets an example which may, with advantage, be followed by other institutions.

## Figures and Tables

**Figure f1:**
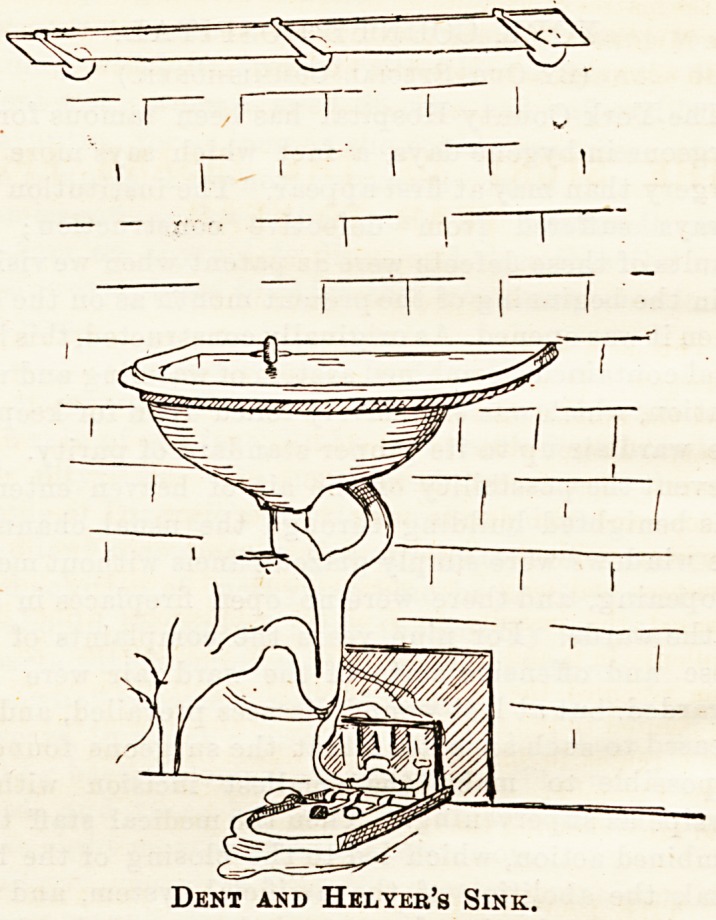


**Figure f2:**